# Prediction of spatiotemporal evolution and zoning of ecological sensitivity in the upper reaches of Minjiang river, sichuan, China

**DOI:** 10.1038/s41598-025-16056-8

**Published:** 2025-08-17

**Authors:** Lingfan Ju, Yan Liu, Shunduo Liu, Qing Xiang, Wenkai Hu, Peng Yu

**Affiliations:** 1 College of Geography and Planning, Chengdu University of Technology, Chengdu, 610059 China; 2 Center for Human Geography of Tibetan Plateau and Its Eastern Slope, Chengdu University of Technology, Chengdu, 610059 China

**Keywords:** CA-MC model, Ecological sensitivity, Geodetector, Minjiang river, Ecology, Environmental sciences

## Abstract

The study of the regional ecological sensitivity evolution process can timely understand the regional ecological evolution law and propose ecological protection strategies. In this study, an ecological sensitivity index system is established to quantitatively analyze the interrelationships of ecological factors. The CA-MC model and center of gravity migration are used to investigate the spatial and temporal evolution of ecological sensitivity in the upper Minjiang River basin from 2000 to 2020 and to predict the ecological sensitivity in 2040. Then with the help of Geodetector to clarify the influence intensity of each factor, different types of ecological regions are identified. The results show that (i) the applicability and accuracy of the analytical framework are verified by categorizing the study area into five classes: insensitive, lightly sensitive, moderately sensitive, highly sensitive, and extremely sensitive. (ii) The ecological sensitivity of the study area from 2000 to 2020 is still dominated by moderately sensitive and highly sensitive. In this process, the environmental protection measures become more and more diversified, and the insensitive areas are increasing. (iii) There is a significant difference in the shift of the center of gravity of ecological sensitivity at all levels, with the overall shift of insensitive, lightly sensitive, and highly sensitive areas to the east. The moderately sensitive and extremely sensitive areas have shifted to the southeast as a whole. Among them, the insensitive area shifted the farthest distance from 2010 to 2020, which was 15.16 km. (iv) The CA-MC model deduced that the overall sensitivity level of the Sichuan-Tibet Railway showed a slow decreasing trend from 2000 to 2040. Through the results of ecological sensitivity evaluation and discussion, this study effectively reveals the differences in ecological sensitivity risks faced by the development of typical watershed areas in China and provides a basis for the government to formulate policies suitable for different environmental protection.

## Introduction

As an increasing number of countries worldwide attach great importance to ecosystem health, ecosystems—within the context of the integrated system of resources, environment, and development—play a crucial role in sustaining human survival and social development^1–3^. As a key component of ecosystem health, scientific evaluation of regional ecological sensitivity is not only essential for rational resource utilization and ecological protection but also a pressing requirement for advancing ecological civilization. Such evaluation results are of great significance for ecological restoration projects, environmental planning, and regional development^4–6^.

Ecological sensitivity refers to the vulnerability and resilience of a region or ecosystem in response to changes and disturbances. It reflects the extent to which ecosystems respond to disturbances from human activities and natural environmental changes, as well as the likelihood of ecological and environmental problems occurring in a region^7^. With the increasing intensity and scope of human activities, their impact on the ecological environment has deepened—for example, through industrial construction and urbanization—leading to changes in land-use types and thus altering regional ecological characteristics^8–10^.

Existing research indicates that it is more common to analyze the effects of geomorphic setting, soil type, vegetation type, land cover, and land use landscape type on ecological sensitivity^11,12^. Elevation, slope orientation, and gradient are key quantitative indicators of geomorphic factors. These factors influence the distribution of ecological sensitivity in regional ecosystems by regulating the distribution of different natural species^13^. Vegetation types directly or indirectly affect, to varying degrees, changes in the abundance of organisms in the ecosystem and thus changes in the ecosystem^14^. At the same time, the vital activities of plant organisms play an extremely important role in the resistance of regional ecosystems to disturbances from external activities^15^. In addition, numerous studies have examined the impact of natural environmental drivers, such as climate change, on ecological sensitivity^16,17^.

The upper reaches of the Minjiang River, a typical ecologically fragile mountainous region, serve as a critical ecological functional zone in China. This area holds significant strategic importance for ecological conservation, playing a pivotal role in safeguarding the ecological security of the Yangtze River Basin and fostering sustainable socio-economic development within the region^18,19^. Characterized by rugged terrain, it is highly prone to geological hazards such as avalanches, landslides, and mudflows. Particularly in arid river valleys, complex geological and natural conditions, coupled with extreme ecological fragility and underdeveloped economies, highlight the need for in-depth research on ecological sensitivity^20,21^.

GIS analysis of watershed ecological sensitivity is an important means of regional ecological research, with the development of machine learning, ecologically sensitive areas are also gradually from the static analysis of hierarchical analysis, cluster analysis, and principal component analysis to the dynamic prediction of artificial neural networks (ANN) and Markov chain (MC), which improves the feasibility of the study and the application of the value, presenting a multi-faceted reveal ecologically sensitive patterns and processes of the steady state characteristics of the ecological sensitivity patterns and processes^22,23^. MC and cellular automata (CA) are both time-discrete and state-discrete dynamical models, and MC model has the advantage of predicting the future land quantity, but it cannot reflect the changes of spatial distribution pattern, and CA model can effectively simulate the interactions between cells, and has strong spatial analysis and simulation ability, but there are limitations^24,25^. The CA-MC coupled model can realize the long-term prediction of the spatial evolution of complex systems and has been widely used in ecological research and achieved good results^26^.

Therefore, the objectives of this study are: (i) to construct an ecological sensitivity index system to grade the ecological sensitivity of the study area; (ii) to analyze the distributional characteristics of the ecological sensitivity center of gravity shift in the study area; (iii) to deduce the spatial evolution of ecological sensitivity in the study area based on the CA-MC model; and (iv) to provide restorative suggestions by zoning the ecological sensitivity of the study area.

## Materials and methods

### Study area

**Fig. 1:**
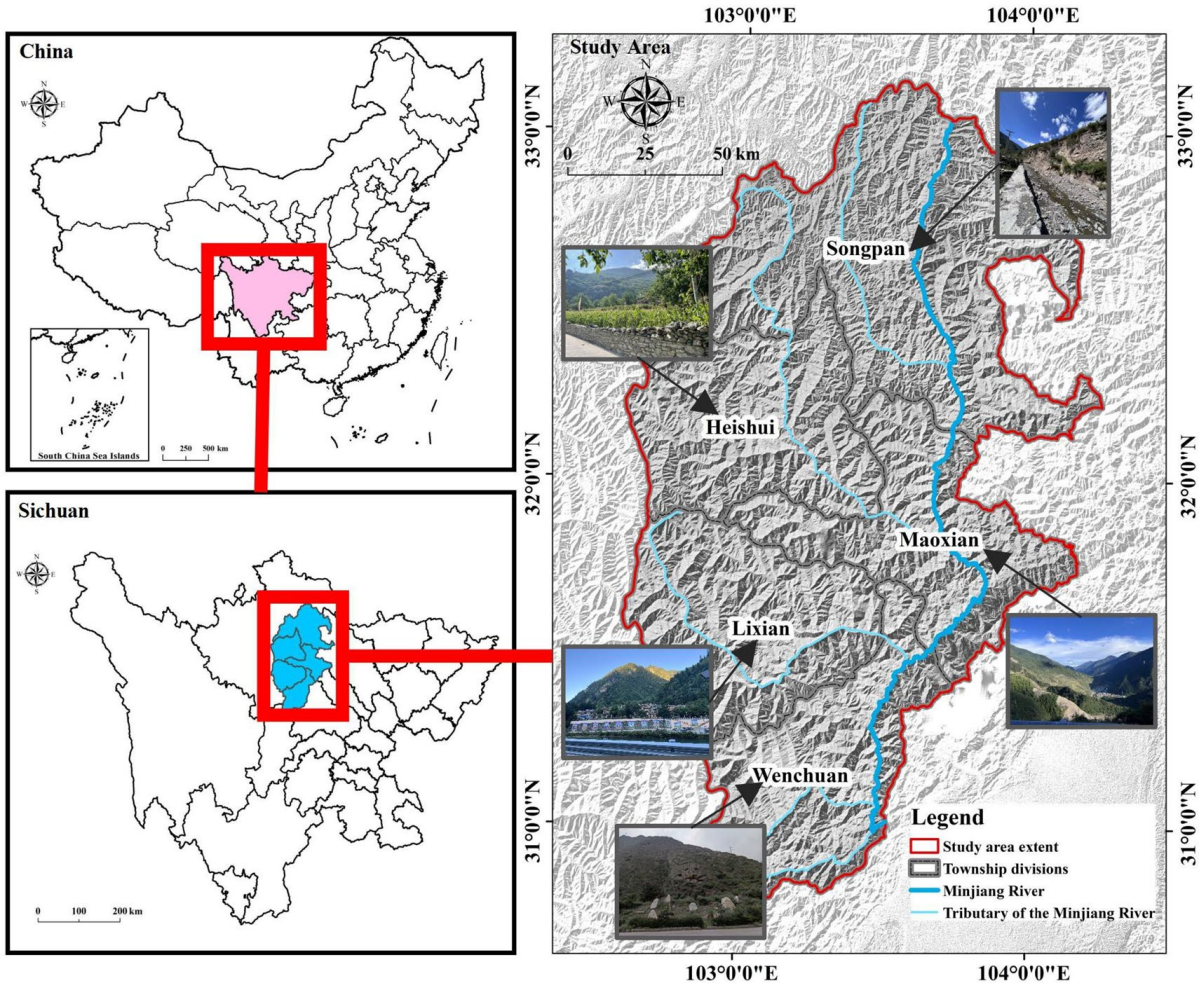
Location of the study area. The map was created using ArcMap (version 10.8, https://www.esri.com/en-us/arcgis/products/index).

The Minjiang River is one of the most important tributaries of the upper reaches of the Yangtze River in China. Its upper basin is located in the high mountain valley zone on the eastern edge of the Tibetan Plateau, at the border between the edge of the Tibetan Plateau and the Chengdu Plain^27^. It roughly coincides with the administrative boundaries of Songpan, Heishui, Maoxian, Lixian, and Wenchuan in the Aba Tibetan and Qiang Autonomous Prefecture of Sichuan Province, covering an area of about 24,742.27km^2^ (Fig. 1). The area is agriculturally developed and subject to urbanization. The development of villages has accelerated, and the industrial and tertiary sectors are developing fast. However, there are environmental problems such as water environment pollution, vegetation degradation, and disasters^18^. Therefore, it is necessary to propose effective management strategies for environmental prevention and control with a full understanding of the spatial and temporal evolution of the ecological sensitivity of the watershed.

### Data sources

The datasets applied in the research include the digital elevation model (DEM), normalized difference vegetation index (NDVI), land use and cover change (LUCC), evaporation, rainfall, population density, stratigraphic lithology, Night-Time light, soil erosion, soil type, and road. Among them, NDVI, LUCC, evaporation, rainfall, Night-Time light, and population belong to the dynamic change indicators. Therefore, their data for 2000, 2010, and 2020 are selected in this study. DEM, stratigraphic lithology, soil erosion, soil, and road are stable indicators, so only one year between 2000 and 2020 is selected as research data in this study. Detailed information on all data is presented in Table 1.

**Table 1:** Data sources.

Data	Resolution	Year	Data Resource
NDVI	30 m	2000–2020	Resource and Environment Science and Data Center (http://www.resdc.cn/, accessed on 26 January 2024)
LUCC	30 m	2000–2020	Resource and Environment Science and Data Center (http://www.resdc.cn/, accessed on 26 January 2024)
Evaporation	1 km	2000–2020	National Tibetan Plateau Scientific Data Center (https://data.tpdc.ac.cn/, accessed on 26 January 2024)
Rainfall	1 km	2000–2020	China Scientific Data Network (https://www.scidb.cn/, accessed on 26 January 2024)
Population	1 km	2000–2020	WorldPop (https://hub.worldpop.org/, accessed on 26 January 2024)
Night-Time Light	1 km	2000–2020	Resource and Environment Science and Data Center (http://www.resdc.cn/, accessed on 26 January 2024)
DEM	30 m	2020	Geospatial Data Cloud (https://www.gscloud.cn/, accessed on 26 January 2024)
Stratigraphic Lithology	1 km	2008	Resource and Environment Science and Data Center (http://www.resdc.cn/, accessed on 26 January 2024)
Soil Erosion	1 km	2010	Resource and Environment Science and Data Center (http://www.resdc.cn/, accessed on 26 January 2024)
Soil	1 km	2009	Resource and Environment Science and Data Center (http://www.resdc.cn/, accessed on 26 January 2024)
Road	1 km	2020	National Catalogue Service For Geographic Information (http://www.webmap.cn/, accessed on 26 January 2024)

### Methods

#### Research framework

**Fig. 2:**
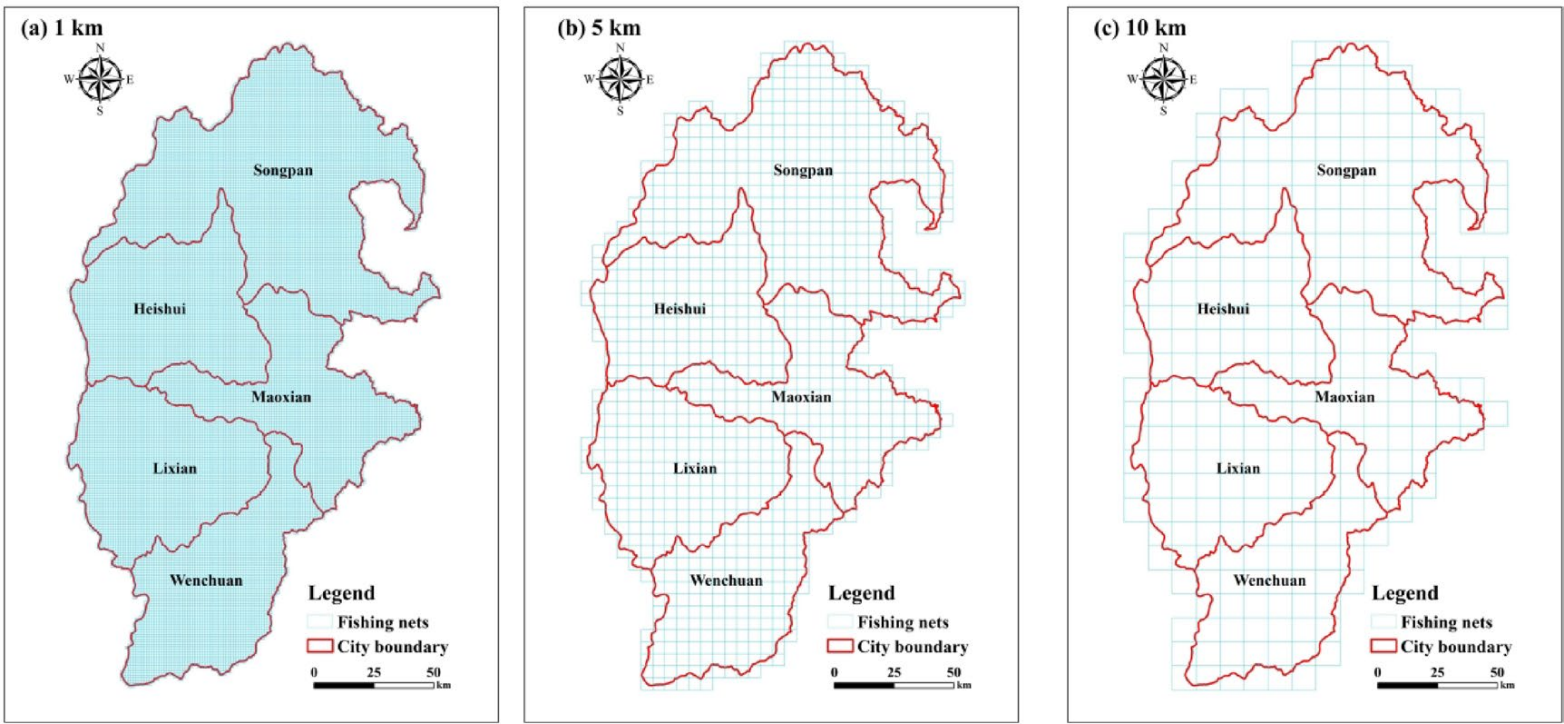
Technical Process.

**Fig. 3:**
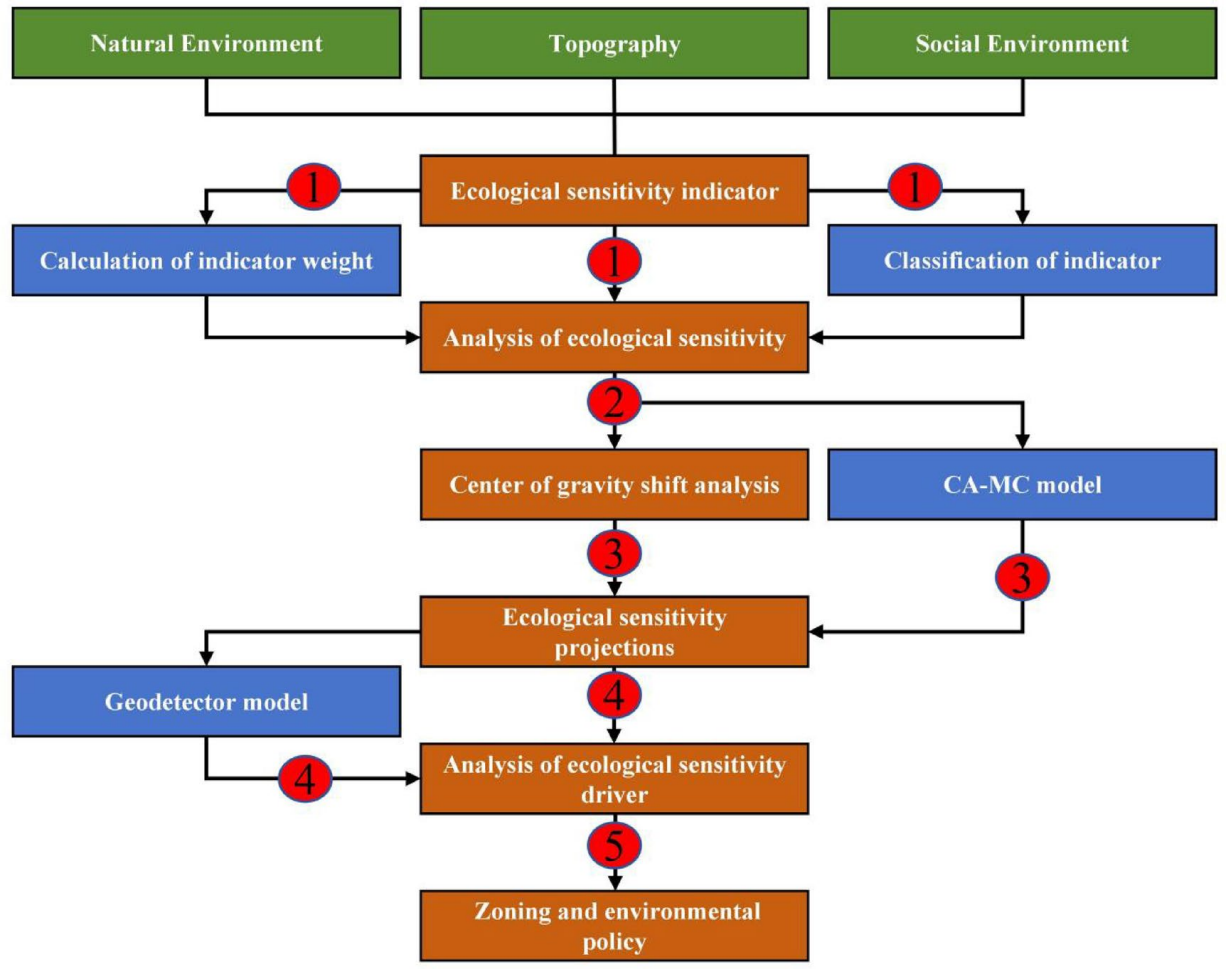
Schematic diagram of fishing nets division in the study area of 1 km (**a**), 5 km (**b**), and 10 km (**c**). The map was created using ArcMap (version 10.8, https://www.esri.com/en-us/arcgis/products/index).

We constructed a research framework to analyze the ecological sensitivity of the study area (Fig. 2). Firstly, 11 ecological sensitivity indicators were selected from the three aspects of “natural environment-geomorphology-social environment”, and the calculation and grading methods of the indicators are shown in Table 2. The indicator factors are spatially and regionally heterogeneous^28^. Therefore, this paper utilizes the fishing net tool of ArcGIS 10.8 software to divide the study area into grid cells of the same scale. After several tests and comparisons (Fig. 3), and referring to related literature^17,29^. The study area was divided into 1 km × 1 km fishing nets, and 25,388 vector data maps with equal amplitude were obtained. The spatial and temporal distribution and evolutionary characteristics of ecological sensitivity in the study area were quantified using the indicator data.

Next, we calculated the weights of the indicators, then used these weights to generate spatiotemporal distribution maps of integrated ecological sensitivity and analyzed the characteristics of the center of gravity shift. Then, the CA-MC model was used to deduce the spatial distribution of ecological sensitivity in the study area in 2040. Finally, Geodetector was used to analyze and interpret the influence of indicator factors on the spatial distribution phenomenon and to partition the area.

The aridity index is the ratio of evaporation to precipitation. The slope was calculated based on DEM data with the help of Arc Map 10.8 software.

**Table 2:** Ecological sensitivity evaluation index system.

Classification	Index System	Ecological sensitivity classification				
		Insensitive	Lightly sensitive	Moderately sensitive	Highly sensitive	Extremely sensitive
Natural environment	NDVI	< 0.2	0.2–0.4	> 0.4–0.6	> 0.6–0.8	> 0.8
	LUCC	Barren, Impervious	Cropland	Grassland	Forest, Wetland, Shrub	Water, Snow/Ice
	Aridity index	< 0.4	0.4–0.6	> 0.6–0.8	> 0.8-1	> 1
Topography	Elevation (m)	< 2000	2000–3000	> 3000–4000	> 4000–5000	> 5000
	Slope (°)	< 8	8–15	> 15–25	> 25–35	> 35
	Soil type	Alisols, Podzoluvisols, Phaeozems	Luvisols, Greyzems	Cambisols, Gleysols	Regosols, Leptosols	Rock outcrops, Water bodies
	Stratigraphic lithology (MPa)	> 60	> 30–60	> 15–30	5–15	< 5
	Soil erosion (mm/a)	< 0.74	0.74–3.7	> 3.7–5.9	> 5.9–11.1	> 11.1
Social Environment	Distance from road (m)	< 200	200–400	> 400–600	> 600–800	> 800
	Population density (person/km^2^)	< 1	1–3	> 3–5	> 5–10	> 10
	Night-Time light	Divided into five levels by using the natural breakpoint method				

#### Impact factor weighting analysis

The degree of dispersion of the indicators is judged by calculating the entropy value, and the greater the degree of dispersion, the greater the influence of the indicator on the comprehensive evaluation^30^. Determine the factor weights according to the relative degree of change of the factors on the overall system, with strong objectivity, the specific steps are: set the data matrix, where *x*_*ij*_ is the value of the *j* indicator in the *i* year, the calculation formula is:

1 $$\:\begin{array}{ccc}A&\:=&\:\left[\begin{array}{ccc}{x}_{11}&\:\cdots\:&\:{x}_{1m}\\\:\vdots&\:\ddots\:&\:\vdots\\\:{x}_{n1}&\:\cdots\:&\:{x}_{nm}\end{array}\right]\end{array}$$

Then, data standardization: the unit of measurement of each indicator is not uniform, data standardization is required; and because the numerical meanings of positive and negative indicators are different, different algorithms are required to standardize the data for positive and negative indicators, the calculation formula is as follows:

2 $$\:{x}_{ij}=\frac{{x}_{ij}-\text{min}\left({x}_{ij}\right)}{\text{max}\left({x}_{ij}\right)-\text{min}\left({x}_{ij}\right)}+0.00001$$

3 $$\:{x}_{ij}=\frac{\text{max}\left({x}_{ij}\right)-{x}_{ij}}{\text{max}\left({x}_{ij}\right)-\text{min}\left({x}_{ij}\right)}+0.00001$$

Calculate the entropy value (*W*_*j*_) of the j indicator: the calculation formula is:

4 $$\:{W}_{j}=\frac{1}{\text{l}\text{n}\left(n\right)}{\sum\:}_{i=1}^{n}\frac{{T}_{ij}}{\sum\:_{i=1}^{n}{T}_{ij}}ln\frac{{T}_{ij}}{\sum\:_{i=1}^{n}{T}_{ij}}$$

where *T*_*ij*_ is the standardized data for year *i* of indicator *j*; and is the dimensionless value of each indicator after data standardization and non-negativity.

#### Spatial center of gravity shift analysis for ecological sensitivity

The planar center of gravity model can reflect the spatial center of gravity migration process of ecologically sensitive areas, and the calculation of the center of gravity position of each ecologically sensitive area in different periods can reveal the spatial evolution process of ecologically sensitive areas in two-dimensional space, and the calculation formula is as follows:

5 $$\:x=\frac{{\sum\:}_{i=1}^{n}({c}_{i}\times\:{x}_{i})}{{\sum\:}_{i=1}^{n}{c}_{i}}$$

6 $$\:y=\frac{{\sum\:}_{i=1}^{n}({c}_{i}\times\:{y}_{i})}{{\sum\:}_{i=1}^{n}{c}_{i}}$$

where *x* and *y* are the latitude and longitude coordinates of the center of gravity of a landscape type, respectively; and *c*_*i*_ is the area of *i* patch of the landscape type; *x*_*i*_ and *y*_*i*_ are the latitude and longitude coordinates of the center of gravity of the distribution of *i* patch of a landscape type, respectively.

#### CA-MC model predictive analysis

CA model consists of 4 parts: cell and its state, cell space, cell neighborhood, and transition rules^31^. It is discrete in time, space, and state. Arbitrary tuple variables exist only in finite and discrete states and are synchronously modified according to the same transition rules. The rules for state change are localized in time and space. The general CA model calculation formula is:

7 $$\:S=\left(t+1\right)=f[S\left(t\right),N]$$

where *S* is the set of finite and discrete states of the tuple; *N* is the neighborhood of the tuple; and *f* is the transition rule of the locally mapped tuple.

The MC model is used to calculate the spatial transition probability and to explain the characteristic law of change transfer of an event in a certain period of time by using the statistics of before and after state development in time. The evolution of ecological sensitivity is characterized by the MC process, and its gradation corresponds to the “possible states” in the MC process, while the area or proportion of mutual transformation between gradations is the state transfer probability, expressed as follows:

8 $$\:{N}_{t+1}={N}_{t}^{\ast\:}{P}_{ij}$$

9 $$\:{P}_{ij}=\left[\begin{array}{ccc}{P}_{11}&\:\cdots\:&\:{P}_{1n}\\\:\vdots&\:\ddots\:&\:\vdots\\\:{P}_{n1}&\:\cdots\:&\:{P}_{nn}\end{array}\right]$$

where *N*_*t+1*_ denotes the ecological sensitivity grading in the later period; *N*_*t*_ denotes the ecological sensitivity grading in the previous period; *P*_*ij*_ denotes the state transfer probability; 0 ≤ *P*_*ij*_<1且$\:{\sum\:}_{j=1}^{n}{P}_{ij}=1(i,j=\text{1,2}\cdots\:,n)$.

The MC model has the ability of time series projection, while the CA model has the advantage of predicting the spatial and temporal dynamics of complex systems, so the combination of the two models can scientifically project the spatial changes of the ecological sensitivity of the watershed^32^.

In this study, the accuracy of the prediction of the evolution of ecological sensitivity was tested by using the Kappa coefficient. It is the result of summing over all categories by multiplying the total number of image elements of all real references by the sum of the diagonals of the confusion matrix, subtracting the product of the number of real references in each category and the total number of categorized image elements in that category, and then dividing the sum by the square of the total number of image elements subtracting the product of the total number of real references in each category and the total number of categorized image elements in that category. The Kappa coefficient takes into account not only the pixels that are correctly categorized on the diagonal, but also various omission and misclassification errors that are not on the diagonal, which can reflect the accuracy of the classification results in a more comprehensive way, and overcomes the limitation that metrics such as the overall classification accuracy only take into account the proportion of the correctly categorized pixels^33,34^. However, the Kappa coefficient is highly sensitive to the marginal distribution of categories, and if the distribution of categories changes, the Kappa value may change significantly even if the performance of the classifier remains unchanged, leading to a lack of comparability across different datasets or tasks^35^. The calculation formula is:

10 $$\:Kappa=({P}_{0}-{P}_{c})/({P}_{p}-{P}_{c})\:$$

where *P*_*0*_ is the proportion of correct simulations; *P*_*c*_ is the proportion of correct predictions in the randomized case of the model; and *P*_*p*_ is the proportion of correct predictions in the ideal case.

#### Geodetector model

The Geodetector is a spatial analysis tool based on data mining and statistical methods aimed at analyzing and explaining the effects of different factors on spatially distributed phenomena, including four modules: factor detection, interaction detection, risk detection, and ecological detection. This study explores the driving factors in the study area based on the factor detection module and interaction detection module. The basic purpose of factor detection is to identify the cause of geographic variation, the explanatory power of the independent variable *x* on the attribute *y* of ecological sensitivity change, and to calculate it using the following equation:

11 $$\:q=1-\frac{\sum\:_{h=1}^{L}{N}_{h}{\sigma\:}_{h}^{2}}{N{\sigma\:}^{2}}=1-\frac{T}{W}$$

12 $$\:W={\sum\:}_{h=1}^{L}{N}_{h}{\sigma\:}_{h}^{2}$$

13 $$\:T=N{\sigma\:}^{2}$$

where *h* = 1,…, *L* is the strata of variable *y* or factor *x*, *N*_*h*_ and *N* are the number of units in layer *h* and the whole area respectively. $\:{\sigma\:}_{h}^{2}$ and $\:{\sigma\:}^{2}$ are the variance of variable *y* in layer *h* and the whole region, respectively. *W* and *T* respectively represent the sum of variance within the layer and the total variance of the whole region.

Interaction tests can quantitatively represent the interaction of two drivers on the distributional pattern of ecological vulnerability, and the types of interaction tests outlined in Table 3 can be used to identify how the drivers interact with each other.

**Table 3:** Types of interaction between explanatory variables.

Interaction	Description
Weakened, nonlinear	q (X1 ∩ X2) < Min (q (X1), q (X2))
Weakened, uni-factor	Min (q (X1), q (X2)) < q (X1 ∩ X2) < Max (q (X1), q (X2))
Enhanced, bi-factors	q (X1 ∩ X2) > Max (q (X1), q (X2))
Independent	q (X1 ∩ X2) = q (X1) + q (X2)
Enhanced, nonlinear	q (X1 ∩ X2) > q (X1) + q (X2)

## Results

### Spatial distribution characteristics of indicators

**Fig. 4:**
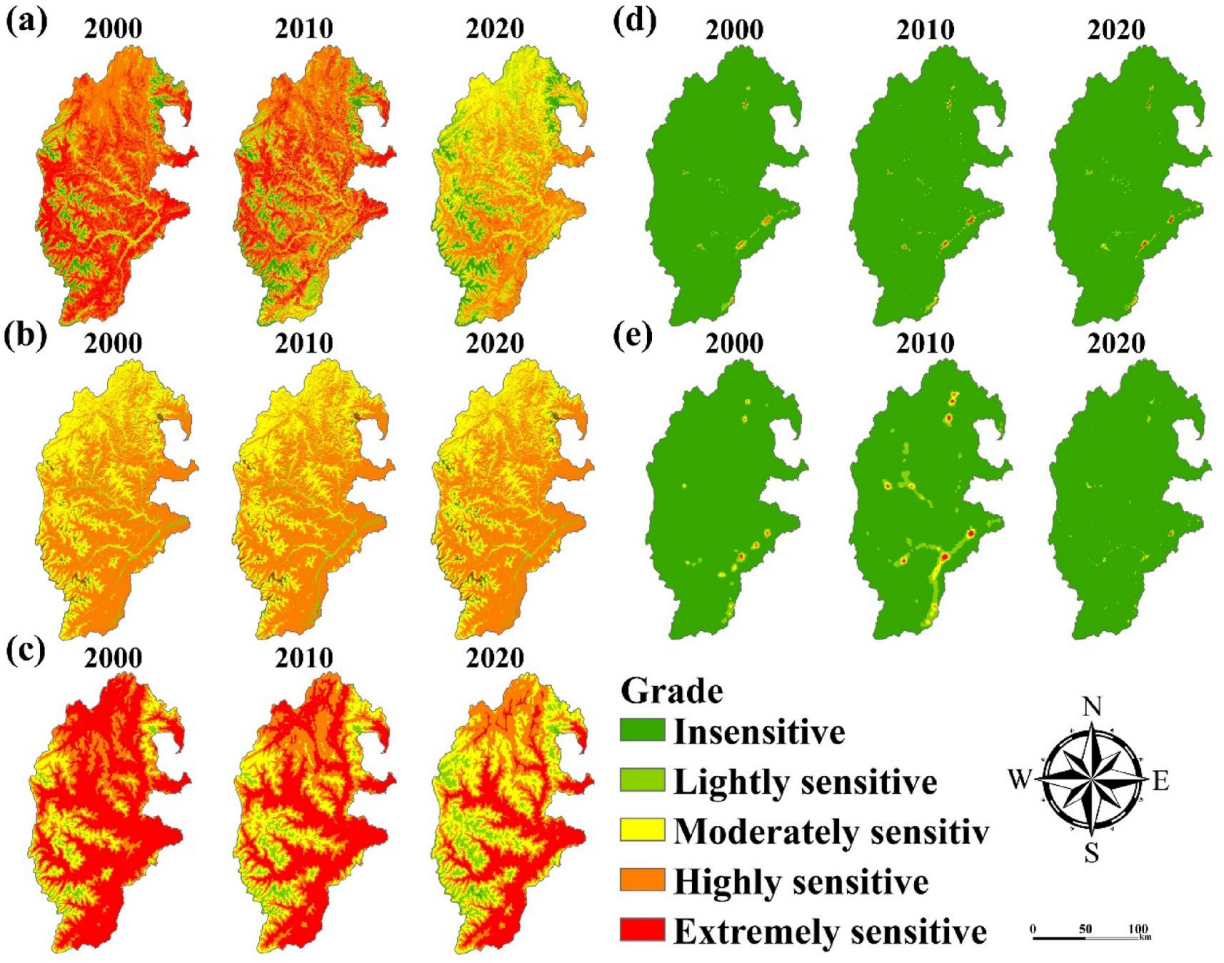
Spatial distribution characteristics of dynamic change indicators (**a**) NDVI, (**b**) LUCC, (**c**) Aridity, (**d**) Population, (**e**) Night-Time light. The map was created using ArcMap (version 10.8, https://www.esri.com/en-us/arcgis/products/index).

**Fig. 5:**
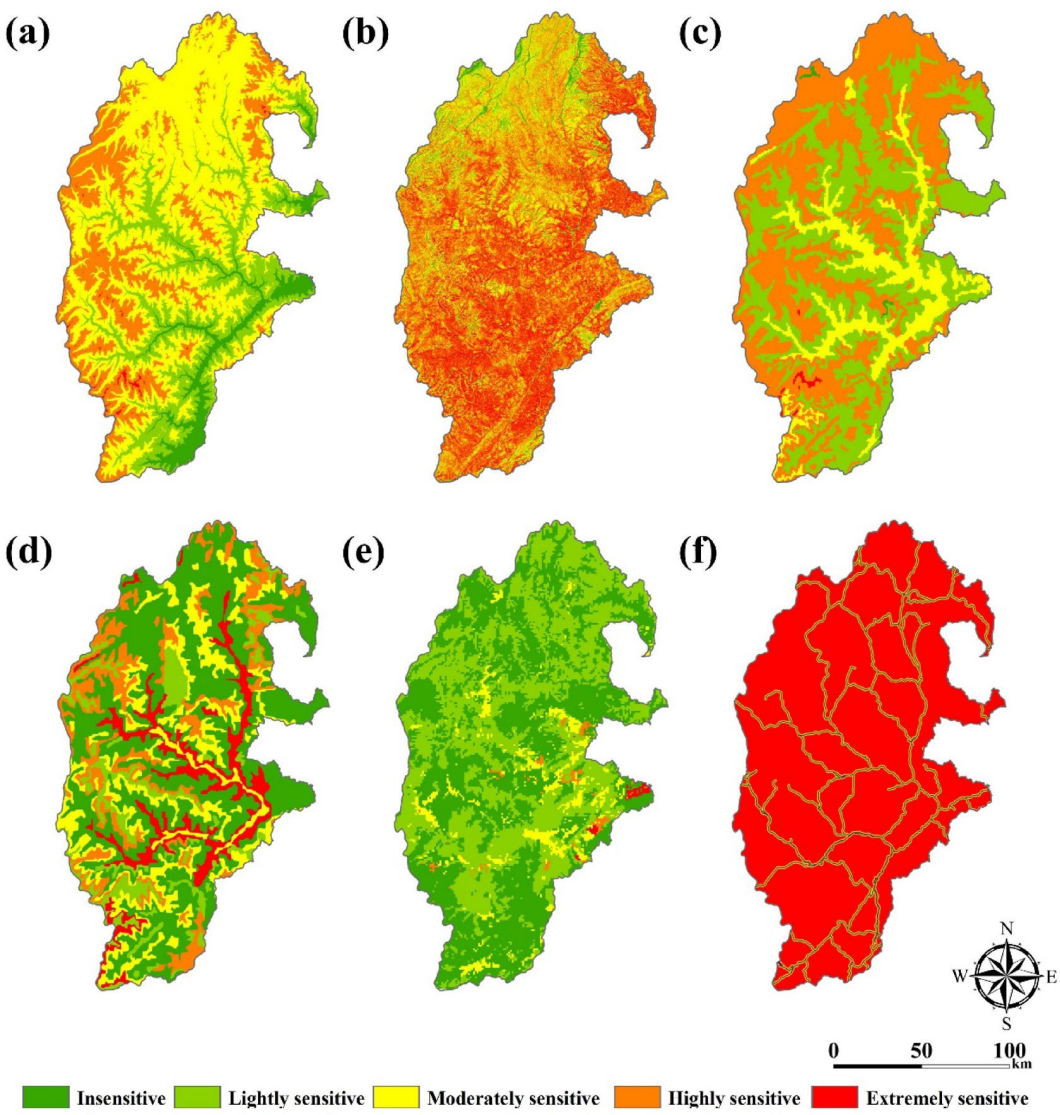
Spatial distribution characteristics of stability indicators (**a**) Elevation, (**b**) Slope, (**c**) Soil type, (**d**) Stratigraphic lithology, (**e**) Soil erosion, (**f**) Distance from road. The map was created using ArcMap (version 10.8, https://www.esri.com/en-us/arcgis/products/index).

According to Table 2, we quantified the indicator data and obtained the spatial distribution of ecological sensitivity of 11 indicator factors. Among them, the spatial distribution characteristics of the dynamically changing indicators are shown in Fig. 4. The characteristics of the spatial distribution of ecological sensitivity of the stabilization indicators are shown in Fig. 5.

From Fig. 4, the spatial and temporal distribution of dynamic indicator factors varies significantly. Among them, NDVI and aridity index changed significantly. There is a significant decrease in the very sensitive areas of the study area.

### Evaluation of ecological sensitivity of integrating factors

**Fig. 6:**
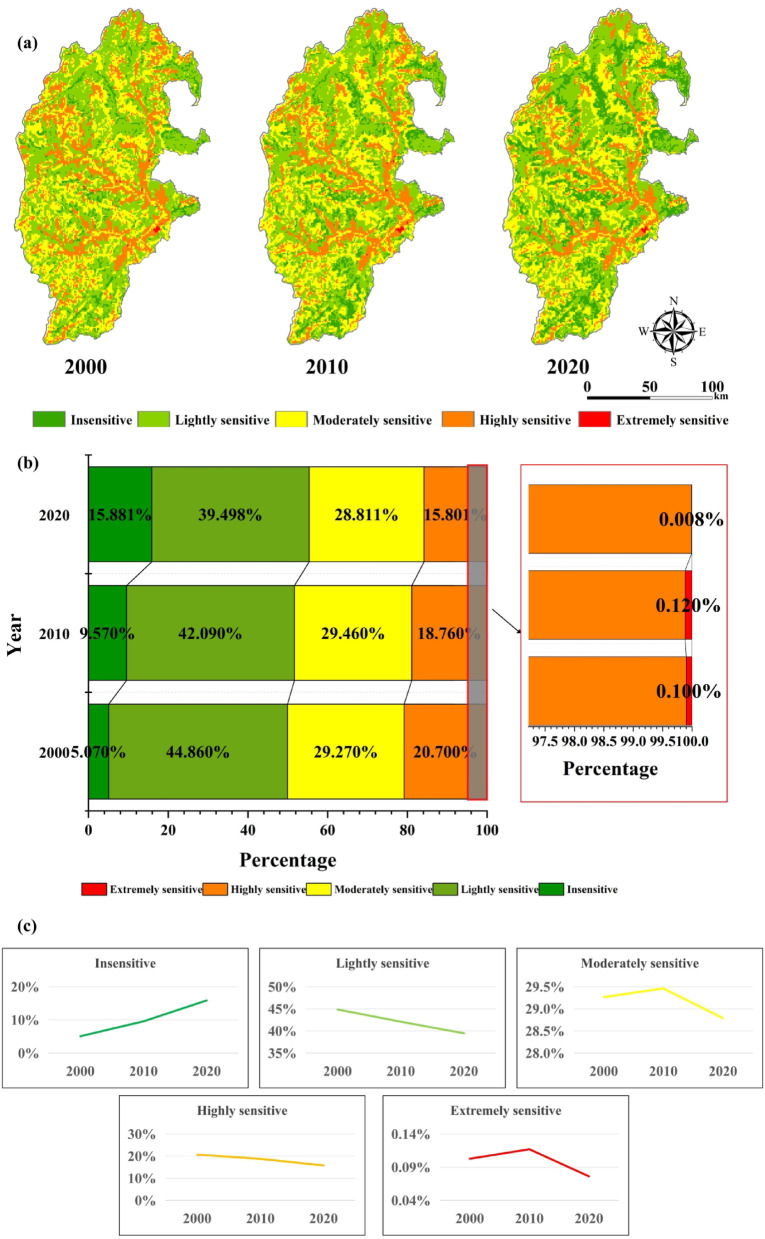
Spatial distribution of integrated ecological sensitivity of study area (**a**), the proportion of ecologically sensitive areas in the study area in 2000, 2010, and 2020 (**b**), and Trends in sensitivity levels (**c**). The map was created using ArcMap (version 10.8, https://www.esri.com/en-us/arcgis/products/index) and Office 365 (Excel, https://www.microsoft.com/).

The above weighting method was used to calculate the factor weights of each indicator (Table 4), and the weighted superposition was calculated to obtain the ecological sensitivity distribution map of the integrated factors in the study area from 2000 to 2020 (Fig. 6a).

**Table 4:** Weight of each factor.

Number	Grade 1	Weight	Grade 2	Weight
1	Natural environment	0.16	NDVI	0.06
			LUCC	0.02
			Aridity index	0.08
2	Topography	0.71	Elevation	0.06
			Slope	0.03
			Soil type	0.1
			Stratigraphic lithology	0.26
			Soil erosion	0.26
3	Social Environment	0.13	Distance from road	0.05
			Population density	0.03
			Night-Time light	0.05

Table 5; Fig. 6b show that the ecological sensitivity of the study area is high from 2000 to 2020, with areas of moderately sensitive, highly sensitive, and extremely sensitive remaining above 50% in 2000 and 2010. It decreased in 2020, accounting for 45% of the study area. Insensitive areas in the study area increased by 10.8%. Overall, the trend of ecological sensitivity has eased. In terms of spatial distribution, the insensitive areas are mostly located on the eastern edge of the study area. Lightly sensitive and moderately sensitive areas were scattered in the study area. Highly sensitive areas are linearly distributed in the central part of the study area. Extremely sensitive areas are mainly concentrated in the eastern part of the study area. In time, the overall trend of sensitivity in the study area is fluctuating and decreasing (Fig. 6c). The area of insensitive area increased by 2602.33km^2^ and the area of highly sensitive and extremely sensitive area decreased by 1186.08km^2^.

**Table 5:** Area and percentage of ecological sensitivity from 2000 to 2020.

Region	2000		2010		2020	
	area (km^2^)	percentage (%)	area (km^2^)	percentage (%)	area (km^2^)	percentage (%)
Insensitive	1221.99	5.07%	2303.72	9.57%	3824.32	15.87%
Lightly sensitive	10801.55	44.86%	10136.55	42.09%	9510.32	39.47%
Moderately sensitive	7047.03	29.27%	7094.82	29.46%	6936.75	28.79%
Highly sensitive	4984.56	20.7%	4518.9	18.76%	3804.88	15.79%
Extremely sensitive	24.75	0.1%	28.18	0.12%	18.35	0.08%

### Ecological sensitivity center of gravity shift analysis

**Fig. 7:**
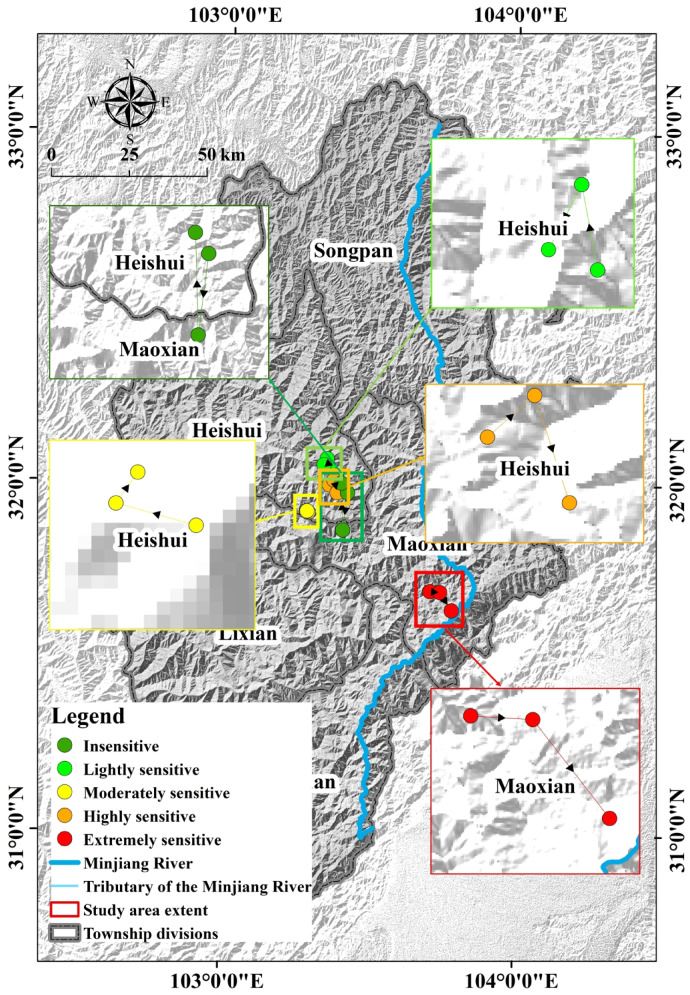
Center of gravity shifts in the sensitive areas of study area from 2000 to 2020. The map was created using ArcMap (version 10.8, https://www.esri.com/en-us/arcgis/products/index).

The center of gravity of the ecologically sensitive areas in the study area shifted from 2000 to 2020, and the sensitive areas shifted to different degrees in the two study periods of 2000–2010 and 2010–2020 (Table 6; Fig. 7). Among them, the insensitive area shifted the farthest distance of 15.16 km in 2010–2020, and the insensitive, lightly sensitive, and highly sensitive areas of the study area shifted to the east as a whole. The moderately sensitive and extremely sensitive areas shifted to the southeast as a whole.

**Table 6:** Center of gravity shift distance in sensitive areas from 2000 to 2020.

Region	Transfer distance/km	
	2000–2020	2010–2020
Insensitive	12.12	15.16
Lightly sensitive	2.57	2.16
Moderately sensitive	0.5	0.26
Highly sensitive	2.13	4.16
Extremely sensitive	3.1	7.04

### CA-MC model integrated ecological sensitivity prediction analysis

**Fig. 8:**
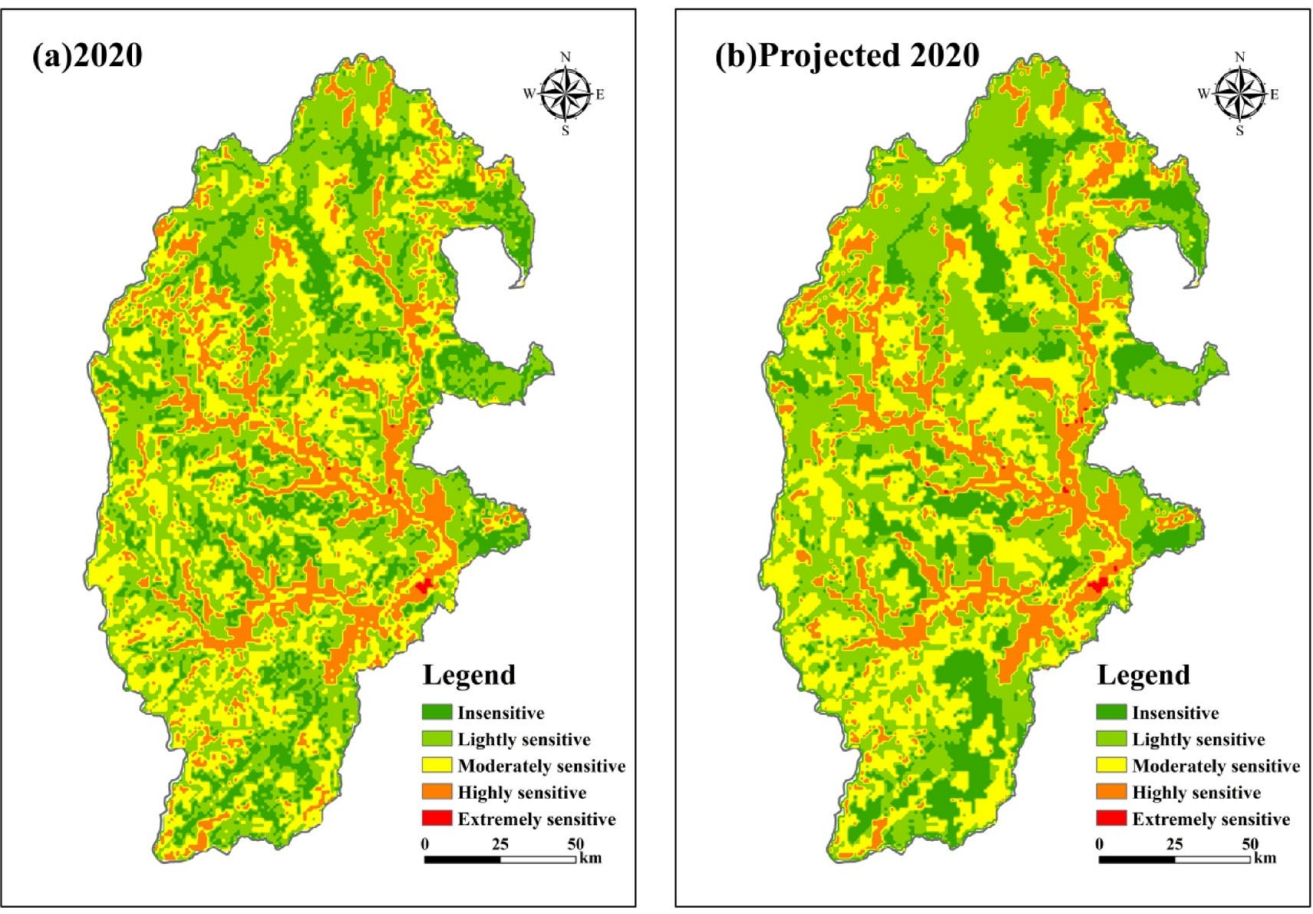
Spatial distribution of integrated ecological sensitivity of study area (**a**), integrated ecological sensitivity projections for the study area in 2040 (**b**). The map was created using ArcMap (version 10.8, https://www.esri.com/en-us/arcgis/products/index).

Based on the CROSSSTAB analysis in IDRISI, the accuracy of the prediction of the ecological sensitivity map and the prediction of each class of the actual ecological sensitivity of the study area in 2020 were calculated (Table 7; Fig. 8). The results show that the insensitive area has the largest error of 236.13 km^2^ and the extremely sensitive area has the smallest error of 10.32 km^2^. According to the formula, the Kappa coefficient of ecological sensitivity of the simulated study area in 2020 is 0.854. Referring to the results of other scholars, the prediction results are better when the Kappa coefficient is greater than 0.75^36–38^. Therefore, the prediction results obtained in this study have good accuracy, which in turn predicts the evolution of ecological sensitivity in the study area in 2040.

**Table 7:** Error between predicted and realistic ecological sensitivity for the study area in 2020.

Region	2020		Projected 2020		Error (km^2^)
	area (km^2^)	percentage (%)	area (km^2^)	percentage (%)	
Insensitive	3824.32	15.87%	3588.19	14.96%	−236.13
Lightly sensitive	9510.32	39.47%	9450.95	39.39%	−59.37
Moderately sensitive	6936.75	28.79%	7000.13	29.18%	63.38
Highly sensitive	3804.88	15.79%	3924.88	16.36%	120.00
Extremely sensitive	18.35	0.08%	28.67	0.12%	10.32

**Fig. 9:**
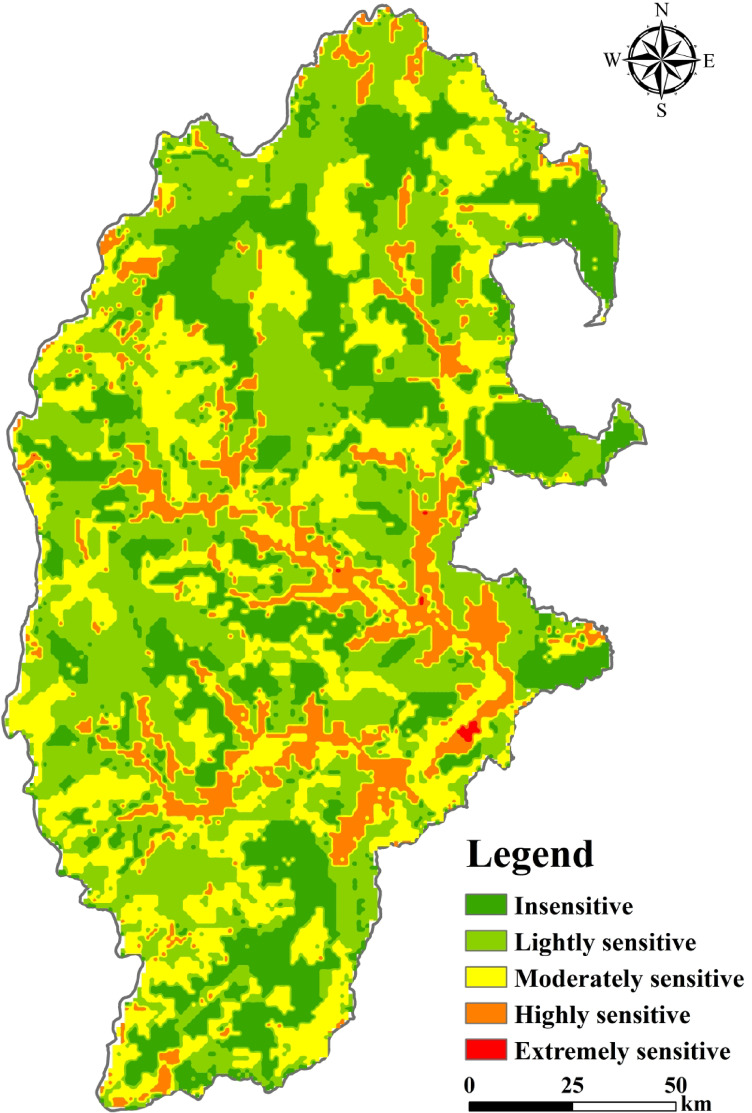
Integrated ecological sensitivity projections for the study area in 2040. The map was created using ArcMap (version 10.8, https://www.esri.com/en-us/arcgis/products/index).

The predicted results of the CA-MC model (Table 8; Fig. 9) show that the sensitivity evolution of the study area in the period of 2000–2020 is similar to that of the period of 2020–2040, and the overall sensitivity level has decreased. By 2040, the proportion of lightly, moderately, and highly sensitive areas continues to decrease by 2.7%, 2.69%, and 4.65%, respectively, shifting to insensitive areas. The insensitive area increases by 10.04% from 2020, with an increase of 2417.68 km^2^. Overall, it reflects that the ecological sensitivity of the study area will decrease from 2020 to 2040.

**Table 8:** Variation characteristics of the study area from 2000 to 2040.

Region	Predicted area in 2040/km^2^	2000–2020		2020–2040	
		Changed area/km^2^	Changed rate/%	Changed area/km^2^	Changed rate/%
Insensitive	6242	2602.33	10.80%	2417.68	10.04%
Lightly sensitive	8858.23	−1291.23	−5.39%	−652.09	−2.70%
Moderately sensitive	6287.24	−110.28	−0.48%	−649.51	−2.69%
Highly sensitive	2684.24	−1179.68	−4.91%	−1120.64	−4.65%
Extremely sensitive	18.47	−6.4	−0.03%	0.12	0

### Analysis of ecological sensitivity drivers

Table 9 shows the results of factor detection, which indicate that soil type (0.1971), stratigraphic lithology (0.1778), aridity index (0.0908), night-time light index (0.0789), soil erosion (0.0702), and population density (0.0608) all have a high level of explanatory power as the key driver of ecological sensitivity, and the rest of the factors have a low level of explanatory power and have little influence on the spatial distribution of ecological sensitivity in the study area.

**Table 9:** Detection table of driving factors of ecological sensitivity.

	Factor	2000		2010		2020		Average	
		q	Number	q	Number	q	Number	q	Number
Environment	NDVI (X1)	0.0199	8	0.0091	9	0.0108	9	0.0133	9
	LUCC (X2)	0.0221	7	0.0242	7	0.0223	7	0.0229	7
	Aridity index (X3)	0.0943	3	0.0923	3	0.0858	3	0.0908	3
Topography	Elevation (X4)	0.0148	9	0.0126	8	0.0168	8	0.0147	8
	Slope (X5)	0.0099	10	0.0082	10	0.0075	10	0.0085	10
	Soil type (X6)	0.2017	1	0.1896	1	0.2	1	0.1971	1
	Stratigraphic lithology (X7)	0.1721	2	0.1696	2	0.1918	2	0.1778	2
	Soil erosion (X8)	0.0594	6	0.0715	5	0.0798	4	0.0702	5
Social Environment	Distance from road (X9)	0.0038	11	0.0034	11	0.0033	11	0.0035	11
	Population density (X10)	0.0679	5	0.0604	6	0.0542	6	0.0608	6
	Night-Time light (X11)	0.0845	4	0.0855	4	0.0667	5	0.0789	4

The results of factor interaction detection are shown in Table 10. Most of the driver factor interactions are bi-enhancement and nonlinear-enhancement there is no nonlinear or independent relationship. The bi-enhancement interaction q-value is greater than the single-factor q-value, indicating that the explanatory power of the two-factor interaction is greater than the single-factor explanatory power, in which the soil type, stratigraphic lithology, soil erosion, and population density have the highest explanatory power in the interaction with the other factors, which further indicates that the soil type, stratigraphic lithology, soil erosion and population density have a greater impact on the spatial distribution of ecological vulnerability in the study area.

**Table 10:** Interaction table of driving factors of ecological sensitivity for 2000, 2010, and 2020.

2020	X1	X2	X3	X4	X5	X6	X7	X8	X9	X10
**X2**	0.02*									
**X3**	0.1**	0.11**								
**X4**	0.03**	0.05**	0.1*							
**X5**	0.02**	0.03**	0.09*	0.02*						
**X6**	0.21*	0.21*	0.23*	0.21*	0.2*					
**X7**	0.21**	0.21*	0.26*	0.21**	0.2**	0.27*				
**X8**	0.1**	0.1**	0.16*	0.1**	0.09**	0.25*	0.25*			
**X9**	0.01*	0.02*	0.09**	0.03**	0.01**	0.2*	0.19*	0.08**		
**X10**	0.06*	0.07*	0.1*	0.07*	0.06*	0.21*	0.24*	0.13*	0.05*	
**X11**	0.08*	0.09*	0.11*	0.09**	0.07*	0.22*	0.26*	0.14*	0.07*	0.08*
Note: * and ** denote bi-enhancement and nonlinear-enhancement, respectively.										

**2010**	**X1**	**X2**	**X3**	**X4**	**X5**	**X6**	**X7**	**X8**	**X9**	**X10**
**X2**	0.03**									
**X3**	0.1*	0.11*								
**X4**	0.04**	0.04**	0.11**							
**X5**	0.02*	0.03**	0.1*	0.02*						
**X6**	0.2**	0.2*	0.23*	0.2*	0.19*					
**X7**	0.19**	0.19*	0.26*	0.19**	0.18**	0.26*				
**X8**	0.09**	0.09*	0.16*	0.09**	0.08**	0.23*	0.22*			
**X9**	0.01**	0.03*	0.1*	0.02**	0.01**	0.19*	0.17*	0.08**		
**X10**	0.07*	0.08*	0.11*	0.07*	0.06*	0.2*	0.22*	0.12*	0.06*	
**X11**	0.09**	0.1*	0.12*	0.1**	0.09*	0.21*	0.24*	0.15*	0.09*	0.09*
Note: * and ** denote bi-enhancement and nonlinear-enhancement, respectively.										

**2000**	**X1**	**X2**	**X3**	**X4**	**X5**	**X6**	**X7**	**X8**	**X9**	**X10**
**X2**	0.03*									
**X3**	0.11*	0.11*								
**X4**	0.05**	0.04**	0.11**							
**X5**	0.03**	0.04**	0.1*	0.02*						
**X6**	0.21*	0.21*	0.25*	0.21*	0.21*					
**X7**	0.19*	0.19*	0.27**	0.19**	0.18**	0.27*				
**X8**	0.08**	0.08*	0.15*	0.08**	0.07**	0.24*	0.22*			
**X9**	0.02**	0.02*	0.1*	0.02**	0.01**	0.2*	0.18*	0.06**		
**X10**	0.09*	0.08*	0.11*	0.08*	0.07*	0.21*	0.24*	0.12*	0.07*	
**X11**	0.1*	0.1*	0.12*	0.1**	0.09*	0.22*	0.25*	0.14*	0.09*	0.1*
Note: * and ** denote bi-enhancement and nonlinear-enhancement, respectively.										

## Discussion

### Spatial and Temporal characteristics of ecological sensitivity

The study of spatial and temporal changes in ecological sensitivity within the regional environment and its prediction quantifies fluctuations in the degree of response of regional ecosystems to natural and human disturbances and reveals the likelihood of the occurrence of potential ecological and environmental problems^39^. It is the basis for ecological environmental protection and provides a scientific basis for the sustainable development of the ecological environment. The study of ecological sensitivity in the upper reaches of Minjiang River is an indispensable part of the ecological and environmental management of the Yangtze River. However, previous research in this area has been limited to the analysis of the current situation, with very limited research on spatial and temporal prediction, a lack of model prediction, and a failure to establish a factor evaluation system for ecological sensitivity applicable to the region. Therefore, understanding the spatial and temporal changes of ecological sensitivity in the region is of great significance for implementing appropriate conservation measures and promoting sustainable development^40^.

The results show high temporal variability and spatial correlation of ecological sensitivity in the study area. It is noteworthy that the eastern smaller blocks exhibit significant spatial clustering of highly sensitive areas, both for the 2000–2020 and projected 2040 results. This is mainly due to the fact that this region is a major urban development area, with most of the land use types in the higher ecological sensitivity category and a higher density of human activities. The ecologically sensitive areas in the central part of the study area are distributed in a strip-like manner, which is related to the fact that the study area belongs to the alpine canyon area, with a V-shaped topography, and human development and construction are carried out along the two sides of the river, which is consistent with the actual situation^41^. The ecologically insensitive areas in the south and north are mainly due to the fact that they belong to the high-altitude area, with a single type of land use and low intensity of human activities, and the ecological environment is also less affected. In this study, Environment, Topography, and Social Environment factors were selected to construct a comprehensive ecological sensitivity evaluation system for the study area. The temporal and spatial evolution characteristics of ecological sensitivity were analyzed, and the entropy value method was used to calculate the weights of the influencing factors, which to a certain extent overcame the characteristics of the AHP hierarchical analysis method and the expert evaluation method, which are more subjective^42^.

The prediction results of ecological sensitivity in the study area show that from 2020 to 2040, the proportions of lightly, moderately, and highly sensitive zones in the region will continue to decrease, all transitioning towards insensitive zones. This is primarily attributed to the improvement of the ecological environment and the increase in ecological protection measures during the period from 2000 to 2020, which led to a significant decline in regional ecological sensitivity^43,44^. Additionally, the local government has introduced and begun implementing a series of ecological and environmental protection policies, which is also one of the main factors contributing to the future decline in ecological sensitivity in the region^45^.

### Center of gravity migration and kernel density in ecologically sensitive regions

The study on the migration of the center of gravity of ecological sensitivity can intuitively reflect the trend of ecological environment change, and at the same time, it can also confirm the center of gravity of the natural environment and human activities. The results of the study show that the overall performance of the center of gravity migration of ecological sensitivity areas in the study area has a large degree of dispersion, and all levels of ecological sensitivity areas have been shifted to different degrees in the process of time evolution. The upper reaches of the Minjiang River, an important tributary of the Yangtze River Basin, are in a special alpine environment, resulting in an ecologically sensitive and extremely fragile environment in the region. In recent years, the region has developed a series of ecological environmental protection measures and large-scale changes in land-use landscape types, which have led to an improvement in the ecology of the region, which is consistent with the results of the study^29,45,46^. As a result, the center of gravity of the ecologically sensitive area has shifted.

### Environmental policy formulation based on factor detection results

**Fig. 10:**
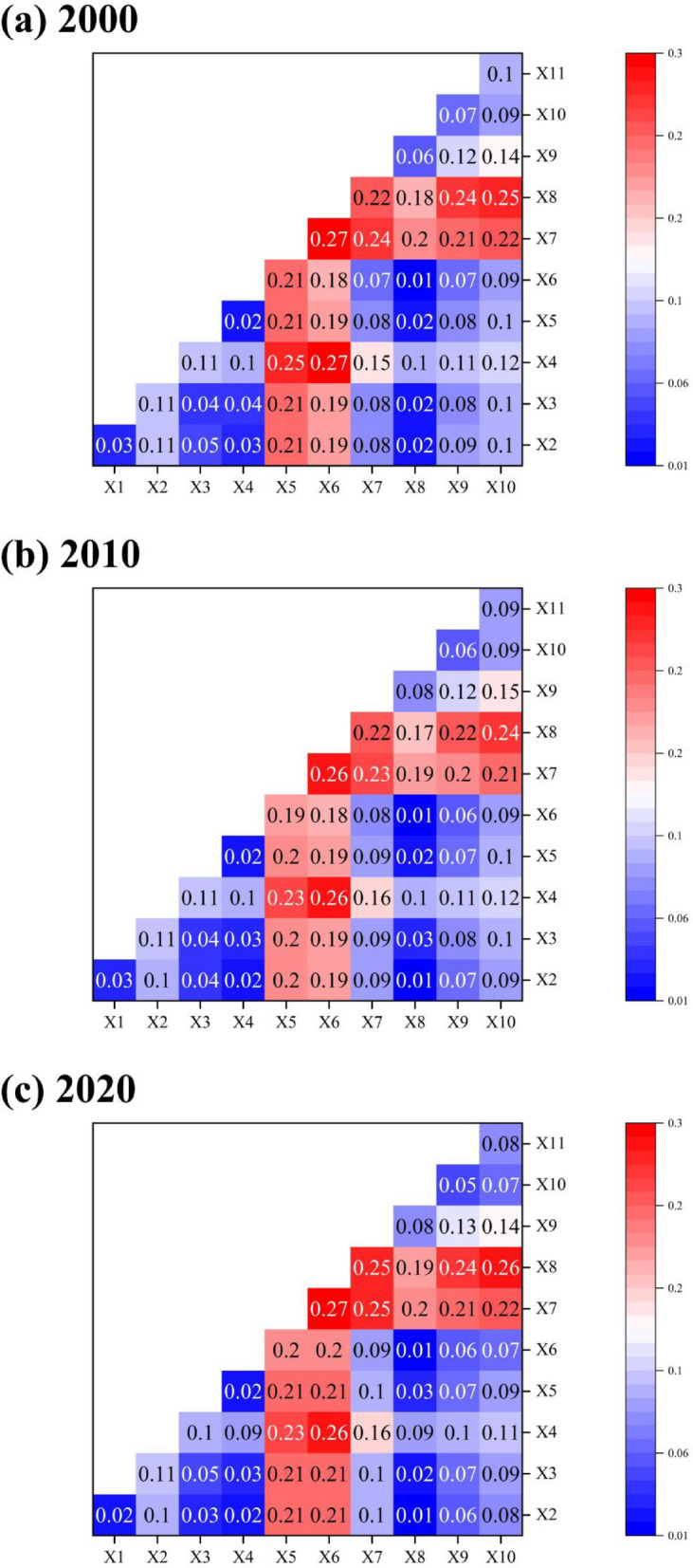
Thermodynamic diagram of driving factor interaction detection.

The empirical results show that factor interaction detection can further clarify the driving causes of ecological sensitivity. To formulate effective environmental policies based on the detection results, we further analyzed the results based on the relevant heat maps (Fig. 10). We found that Elevation (X4), Slope (X5), Soil type (X6), Stratigraphic lithology (X7), and Soil erosion (X8) are the main influential factors. This is because the study area belongs to the alpine valley region with complex topography and east-west undulation. The area is also a typical farming, agro-pastoralist area, where soil and soil texture affect the economic and construction development of the area and influence ecological changes^41^. Compared to the hilly areas of the coast and plains, the study area is influenced by altitude and climate, and its ecological status is not overly dependent on the impact of land changes and anthropogenic economic development. Instead, it is more dependent on the soil and water characteristics resulting from the original climate and topography^37,47,48^.

**Fig. 11:**
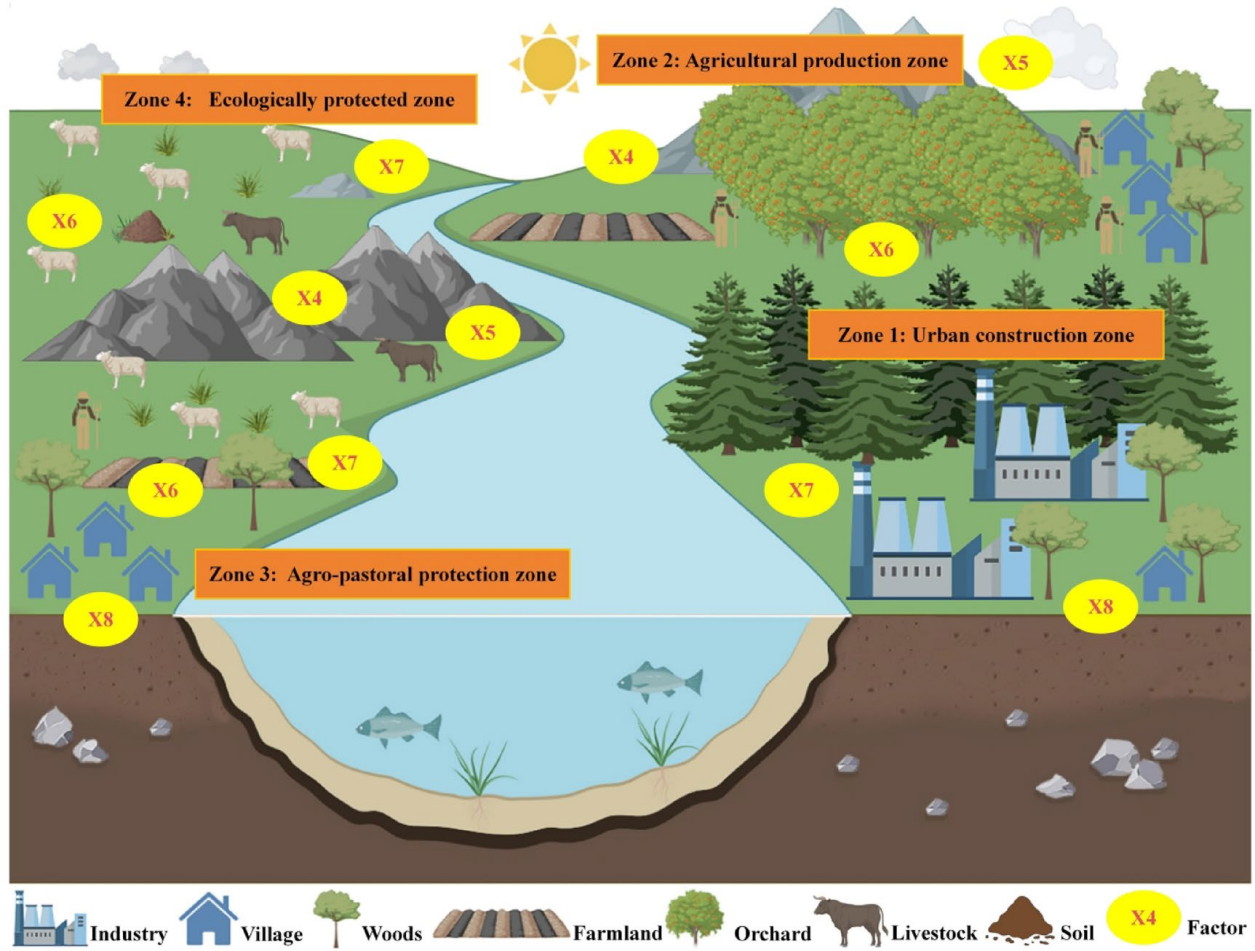
Ecological zoning control policy mapping. The map was created using Office 365 (PowerPoint, https://www.microsoft.com/).

Zone 1 is the urban construction zone (Fig. 11). It is mainly located in the valley area in the central and eastern parts of the study area. Towns and cities are areas of population concentration, accompanied by high-intensity human activities, and they perform the function of economic development. At the same time, industry is its main economic production activity. Therefore, land development and planning should be rationalized in the region, focusing on environmental factors such as soil erosion and soil texture, and environmental monitoring and regulation should be strengthened to prevent environmental degradation^6^.

Zone 2 is the agricultural production zone. It is mainly located in the north and south of the study area. Agriculture is the main mode of production in most parts of the study area, which has become an important way of economic development in the region, with rich resources of arable and orchard land. Elevation, slope, and soil type, which are important factors in agricultural production, determine the growth and development of crops. Therefore, monitoring of agricultural pollution should be strengthened in the region, especially from agricultural production such as fertilizer use and agricultural wastewater. By reducing exogenous emissions of pollutants, soil damage to the region can be effectively reduced and the pressure on ecological sensitivity lowered^49^.

Zone 3 is an agro-pastoral protection zone. It is mainly located in the northern part of the study area in the high altitude grassland areas with gentler terrain. The study area is a multi-ethnic settlement area, with Tibetan populations distributed in mixed nomadic pastoralism and cropland production. Grassland is a moderately ecologically sensitive land use type, vulnerable to the natural environment and human grazing activities. In terms of the influencing factors, soil type, soil texture, and soil erosion are the main environmental factors constraining this area, and the trend of soil desertification is serious. Therefore, it is essential to rationally plan grazing areas, control the number of livestock, strengthen regular soil sampling and monitoring mechanisms, and implement the conversion of farmland to pasture in this region to prevent further desertification of the soil^50^.

Zone 4 is an ecological protected zone. It is mainly located in the western part of the study area in the high-altitude ecological protection zone. There are many nature reserves distributed in the study area with rich species resources and a good ecological environment. Therefore, environmental monitoring and protection should be strengthened in the region to avoid the impact of external sources of pollution on the area and to develop the eco-tourism industry to protect the natural ecological environment^28^.

## Conclusion

This study established an ecological vulnerability evaluation system based on the “natural environment-geomorphology-social environment” and utilized the CA-MC model and Geodetector to evaluate the ecological sensitivity of the study area and the related impact factors and to identify the types of zoning. The main conclusions are as follows: (i) From 2000 to 2020, the trend of ecological sensitivity in the study area has eased, the insensitive area has increased significantly, and the moderately, highly, and extremely highly sensitive areas still dominate. (ii) The center of gravity of the ecological sensitivity in the study area has been shifted to a different degree, among which the insensitive area has shifted the farthest distance of 15.16 km from 2010 to 2020. (iii) The prediction results show that the proportion of lightly sensitive, moderately sensitive, and highly sensitive areas will continue to decrease in 2040, which reflects that the ecological sensitivity of the study area will be reduced in the period from 2020 to 2040. (iv) The study area is classified into 4 types: urban construction zone, agricultural production zone, agro-pastoral protection zone, and ecologically protected zone.

## Data Availability

The data presented in this study are available on request from the corresponding author.
